# Coronavirus Disease 2019 (COVID-19) Associated Hemolytic Uremic Syndrome in a Toddler

**DOI:** 10.1155/2022/3811170

**Published:** 2022-11-23

**Authors:** Kristie Searcy, Apoorva Jagadish, Patricia Pichilingue-Reto, Radhakrishna Baliga

**Affiliations:** ^1^Department of Internal Medicine and Pediatrics, LSU Health Shreveport, Shreveport, LA, USA; ^2^Department of Pediatrics, LSU Health Shreveport, Shreveport, LA, USA; ^3^Department of Pediatrics, Division of Infectious Diseases, LSU Health Shreveport, Shreveport, LA, USA; ^4^Department of Pediatrics, Division of Nephrology, LSU Health Shreveport, Shreveport, LA, USA

## Abstract

Coronavirus disease 2019 (COVID-19) is a heterogenous, predominantly pulmonary disease caused by severe acute respiratory syndrome coronavirus 2 (SARS-CoV-2) that has resulted in catastrophic illness around the world. Thrombotic microangiopathy (TMA) is a triad of hemolytic anemia, thrombocytopenia, and end organ damage. This is present in severe cases of COVID-19 and in hemolytic uremic syndrome (HUS) commonly caused by *Escherichia coli (E.coli)* 0157:H7. We report a novel case of a toddler who presented with classic features suggestive of HUS characterized by bloody diarrhea followed by thrombocytopenia, hemolytic anemia, and acute kidney injury, in whom a polymerase chain reaction (PCR) test for SARS-CoV-2 was positive.

## 1. Case Description

A 2-year-old healthy Caucasian female with no preexisting condition presented with a 7-day history of diarrhea that turned bloody 2 days before admission. Prior to the onset of diarrhea, several close family members developed predominantly upper respiratory symptoms and tested positive for SARS-CoV-2 by PCR. On examination, her weight was 17.1 kg, temperature was 36.1°C, blood pressure (BP) was 137/85 mmHg (>99^th^ percentile), and heart rate was 112/minute. She appeared jaundiced with generalized edema. By day two of admission, diarrhea subsided, urine output decreased considerably, and she appeared fatigued, listless, and irritable. Pertinent laboratory data are provided in Table 1. Laboratory results obtained on admission included the following: Hb: 5.1 g/dL, platelets: 50 k/*μ*L, LDH: 833 U/L, and creatinine: 0.71 mg/dL. Stool culture was negative for *E. coli* 0157: H7 including other enteric pathogens associated with HUS like *Salmonella*, *Shigella*, *Vibrio*, and *Campylobacter*. Shiga toxins 1 and 2 were negative by the enzyme immune assay (EIA). Her nasopharyngeal swab was positive for SARS-CoV-2 by PCR. Complement and ADAMSTS13 levels were normal. A chest radiogram revealed prominent perihilar markings. An echocardiogram showed mild mitral valve stenosis with left atrial enlargement but a normal systolic function. She was provided supportive care, and her blood pressures controlled with labetalol at a dose of 3 mg/kg/day divided twice a day. On day 5, her clinical symptoms including kidney function had improved significantly with a serum creatinine (Cr) of 0.55 mg/dl (eGFR 81 ml/min/1.73 m^2^). She was discharged the following day on labetalol. Four months after discharge, her BP was 107/73 mm Hg (>95th percentile) and serum Cr was 0.32 mg/dL (eGFR 127 ml/min/1.73 m^2^). Her urinalysis showed trace protein and was negative for blood, with a urine protein to Cr ratio of 0.25 g/mmol (Table 1). Her repeat echocardiogram showed a normal systolic function with no mitral valve stenosis or atrial enlargement. Additionally, labetalol was discontinued, and she was started on enalapril at a dose of 0.08 mg/kg given once a day, which was discontinued two months later as her BP normalized being 97/53 mmHg (75^th^ percentile). About nine months after discharge, she had a genetic renal panel (Genetic Renal Panel v8 from Iowa Molecular Otolaryngology and Renal Research Laboratories), which tested for the following 13 genes: CFH, CFI, MCP (CD46), CFB, CFHR5, C3, THBD, DGKE, PLG, ADAMTS13, MMACHC, G6PD, and WT1; this was reported to be negative for any mutations, thus ruling out genetic causes of complement mediated atypical HUS (aHUS). Currently, the patient is off medication and she has a BP of 94/48 mm of Hg (60^th^ percentile), a serum Cr of 0.28 mg/dL; her urinalysis is negative for protein and blood with a urine protein to Cr ratio of 0.11 g/mmol.

## 2. Discussion

The clinical entity of HUS encompasses a group of unique disorders that includes typical HUS, and atypical HUS (aHUS) which results from genetic alteration of the complement mediated or complement independent pathway[1]. Although SARS-CoV-2 primarily targets the lungs and *E. coli* 0157: H7 and Shiga toxins primarily target the kidneys, the pathogenesis of COVID-19 and HUS share similarities as both conditions cause severe endothelial dysfunction resulting in the release of cytokines and inflammatory factors, complement dysregulation, and development of TMA [1, 2]. Both clinical conditions involve cell membrane receptors for cell entry, mainly angiotensin converting enzyme 2 (ACE-2) for SARS-CoV-2 and glycosphingolipid globotriaosylamide (Gb3Cer) for Shiga toxin-associated HUS (ST HUS) [1, 3]. In both COVID-19 and ST HUS, the activation of the renin angiotensin system (RAS) with excessive production of angiotensin can lead to the development of TMA, thus modulating the clinical course of the disease [4, 5]. Our patient presented with features of classic HUS; however, stool culture was negative for *Salmonella*, *Shigella*, *Vibrio*, *Campylobacter*, and *Escherichia coli* 0157: H7. Also ,*E. coli* Shiga toxins 1 and 2 were negative, while the nasopharyngeal swab was positive for SARS-CoV-2.

Recent reports suggest COVID-19 as a potential trigger of aHUS in patients with known genetic complement alteration [6], in those with significant kidney involvement and genetically mediated complement defects [7, 8], and in patients with severe protracted disease who improved significantly with administration of a C5 inhibitor (Eculizumab) [9, 10]. Eculizumab, a monoclonal antibody, has been the mainstay of treatment for aHUS; however, recent data suggest that its benefit as a rescue drug in severe cases of COVID-19 is not clear [11, 12]. Our patient, however, had normal complement levels and genetic testing for complement mediated aHUS was unremarkable. Furthermore, she had complete recovery of kidney function only with supportive treatment which precluded us from performing any further genetic testing at this time.

## 3. Conclusion

Children with COVID-19 typically have a milder kidney disease than that experienced by adults [13]. This report documents COVID-19 in a child who presented with features of HUS. COVID-19 should be considered in the differential diagnosis for any child who presents with bloody diarrhea, consumptive coagulopathy, and acute kidney injury (AKI). It is important to note that this patient had negative genetic markers for complement dependent aHUS and she recovered completely. Careful monitoring post-AKI is crucial to prevent long-term adverse consequences.

## Figures and Tables

**Table 1:** Pertinent laboratory data on admission.

Hematology	Admission values (reference range)	Outpatient values (reference range)
White blood cells (K/*μ*L)	8.1 (6–17.5)	11.68
Hemoglobin (g/dL)	5.1 (10.5–13.5)	11.3
Platelets (K/*μ*L)	51 (150–350)	450
Reticulocyte (%)	8.4% (0–2.5%)	1.2
Peripheral smear	Schistocytes	
Coombs	Negative	
*Coagulation*		
D-dimer (ng/mL)	4098 (<500)	
Haptoglobin (mg/dL)	<8 (30–250)	134
Lactate dehydrogenase (U/L)	833 (100–260)	300
PT	10.4 (9.9–13.4 sec)	
aPTT	29.4 (24.0–39.2 sec)	
*Urine*		
Color	Amber	Yellow
Specific gravity	1.020 (1.005–1.030)	1.025
Protein	3+	Trace
Blood	3+	Negative
Microscopic (/hpf)	8 RBC (0–4/hpf), 21 WBC (0–5/hpf)	5 RBC, 2 WBC
Protein/creatine ratio (g/mmol)	5.1 (<0.2)	0.25
*Inflammatory and cardiovascular parameters*		
Ferritin (ng/mL)	447 (16–300)	207
ESR (mm/h)	25 (0–20)	
Interleukin-6 (pg/mL)	3.0 (≤1.8)	
NT-pro BNP (pg/mL)	3685 (<125)	
*Chemistry*		
Serum creatine (mg/dL)	0.71 (0.2–0.4)	0.32
eGFR (ml/min/1.73 m^2^)	67 (89–165)	127
BUN (mg/dL)	47 (5–18)	10
AST (U/L)	62 (10–40)	45
C3 (mg/dL)	106 (50–180)	
Albumin (g/dL)	3.4 (3.2–4.7 g/dL)	3.9
ANA	Negative	
ADAMSTS13 (%)	>100	
